# Supplementary material for: 5-Aza-2′-Deoxycytidine (5-Aza-dC, Decitabine) Inhibits Collagen Type I and III Expression in TGF-β1-Treated Equine Endometrial Fibroblasts
Source: Animals (Basel). 2023 Mar 30;13(7):1212. doi: 10.3390/ani13071212 (PMC10093662; doi:10.3390/ani13071212)
Supplement: Supplementary file 1 [file animals-13-01212-s001.zip › animals-2222374-supplementary.pdf]

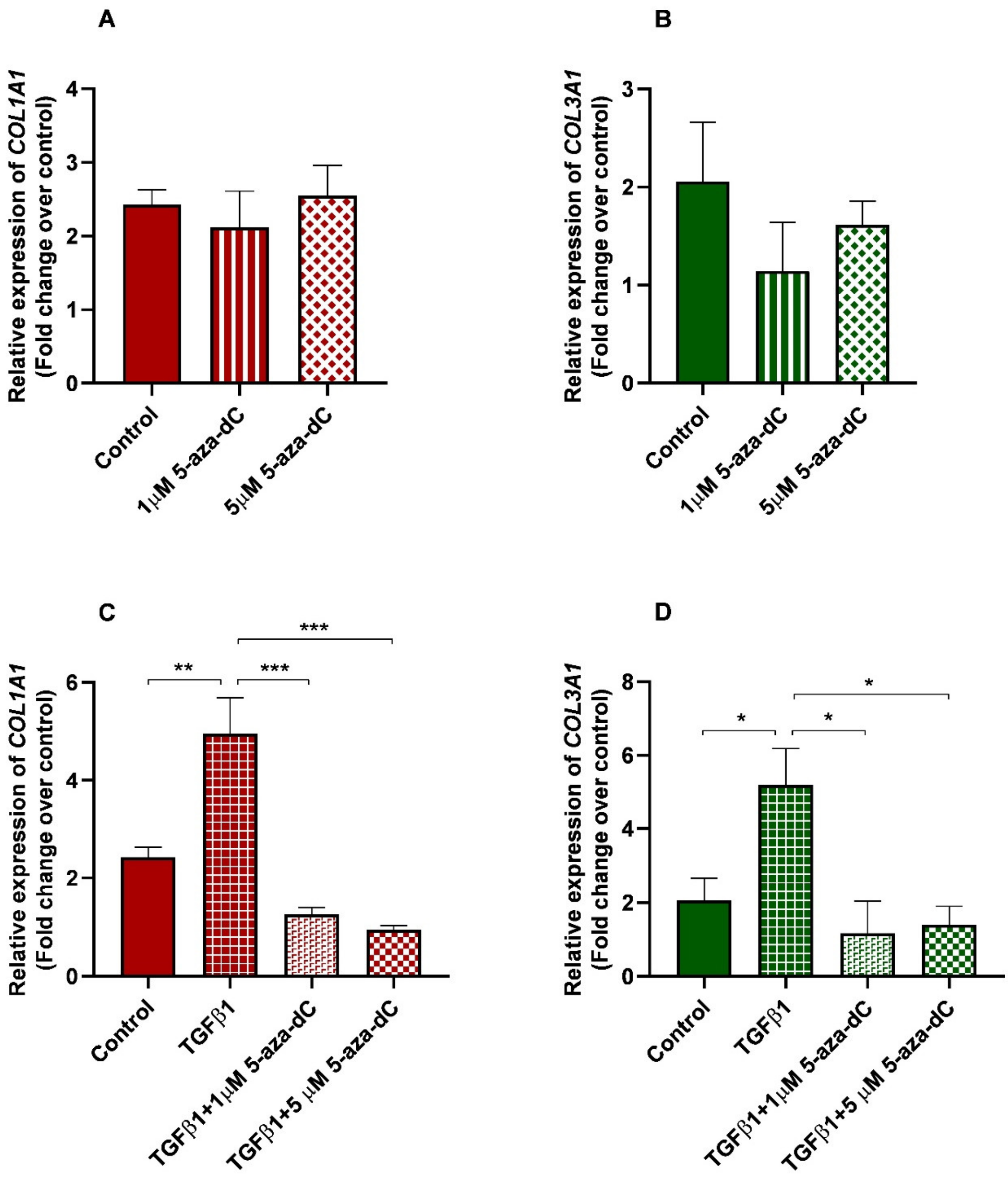

Figure S1. Relative levels of **A)** collagen type I (*COL1A1*), **B)** collagen type III (*COL3A1*) mRNA in endometrial fibroblasts treated with 1μM, 5μM of 5-aza-dC or untreated fibroblasts (control), **C)** collagen type I (*COL1A1*) and **D)** collagen type III (*COL3A1*) mRNA in non-treated (control) endometrial fibroblasts or treated with TGF-β1 (10ng/mL), (1μM), TGF-β1 (10ng/mL) + 1μM 5aza-dC or TGF-β1 (10ng/mL) + 5μM 5aza-dC; n=4. Bars represent mean±SEM. Asterisks indicate significant differences between treatments (*P<0.05, ** P<0.01, *** P<0.001).

1st Protocol

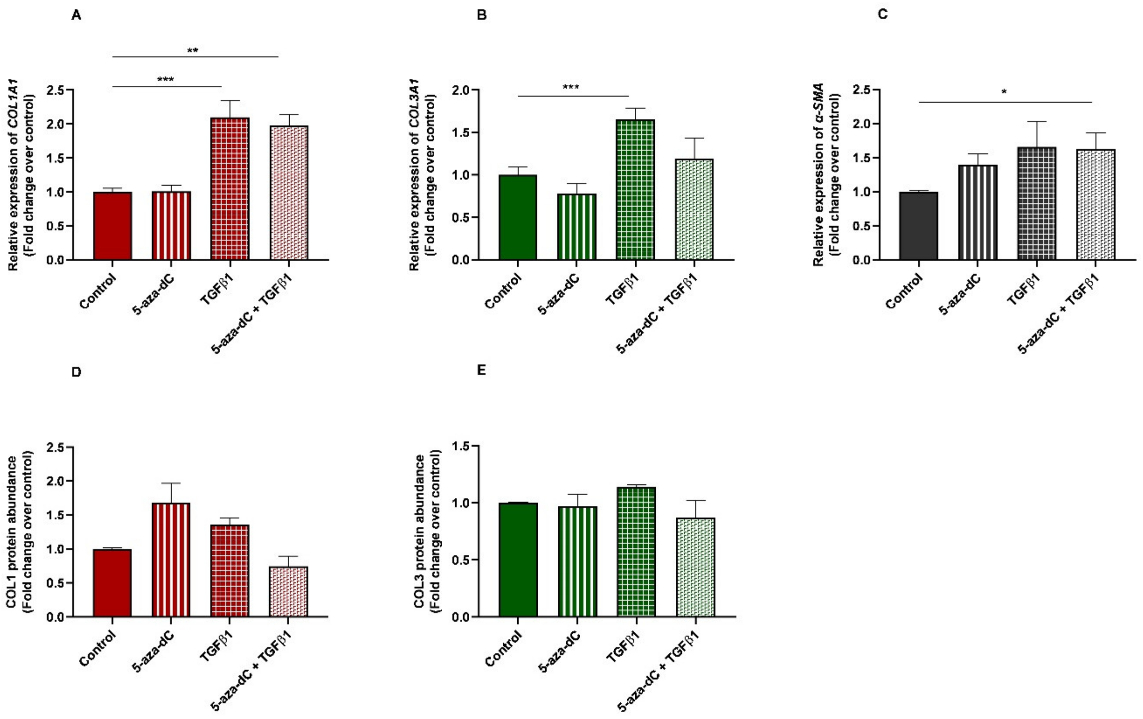

Figure S2. Relative levels of **A)** collagen type I (*COL1A1*), **B)** collagen type III (*COL3A1*), and **C)** α-smooth muscle actin (*α-SMA*) mRNA, and of **D)** COL1 and **E)** COL3 protein concentrations in non-treated (control) endometrial fibroblasts or treated with 5-aza-dC (1μM), TGF-β1 (10ng/mL) or TGF-β1 (10ng/mL) + 5-aza-dC (1μM) for 48h. Each treatment was compared to respective control (all groups with control C and TGF-β1+5-aza-dC with TGF-β1); *n*=5. Bars represent mean±SEM. Asterisks indicate significant differences between treatments (**P*<0.05, ** *P*<0.01, *** *P*<0.001).

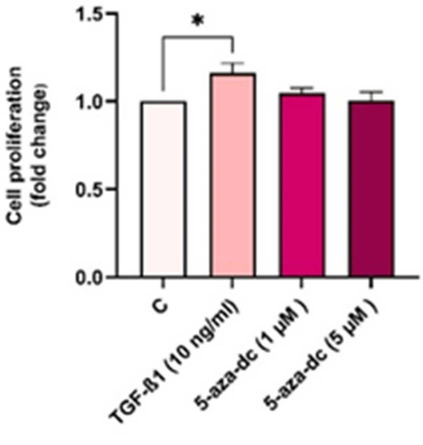

Figure S3. Effect of TGF-β1 at a dose of 10 ng/mL and 5-aza-dC at doses of 1 μM and 5 μM on cell viability¸ *n*=4. Bars represent mean±SEM. Asterisks indicate significant differences between treatment (**P*<0.05). C-control (no factors added to the culture medium).
